# Periventricular Lesions Help Differentiate Neuromyelitis Optica Spectrum Disorders from Multiple Sclerosis

**DOI:** 10.1155/2014/986923

**Published:** 2014-02-09

**Authors:** Eytan Raz, John P. Loh, Luca Saba, Mirza Omari, Joseph Herbert, Yvonne Lui, Ilya Kister

**Affiliations:** ^1^Department of Radiology, New York University School of Medicine, 660 First Avenue, New York, NY 10026, USA; ^2^Department of Radiology, Azienda Ospedaliero Universitaria, di Cagliari Polo di Monserrato, SS 554 Monserrato, 09045 Cagliari, Italy; ^3^NYU Multiple Sclerosis Center, Department of Neurology, New York University School of Medicine, 240 East 38th Street, New York, NY 10026, USA

## Abstract

*Objective.* To compare periventricular lesions in multiple sclerosis (MS) and neuromyelitis optica spectrum disorders (NMOsd). *Materials and Methods.* Sagittal and axial fluid attenuated inversion recovery (FLAIR) sequences of 20 NMOsd and 40 group frequency-matched MS patients were evaluated by two neuroradiologists. On axial FLAIR, periventricular area was characterized as free of lesions/smooth-bordered (“type A”) or jagged-bordered (“type B”) pattern. On sagittal FLAIR, the images were evaluated for presence of “Dawson's fingers.” *Results.* Type A pattern was observed in 80% of NMOsd patients by Reader 1 and 85% by Reader 2 but only in 5% MS patients by either Reader. Type B was seen in 15% NMOsd patients by Reader 1 and 20% by Reader 2 and in 95% MS patients by either Reader. Dawson's fingers were observed in no NMOsd patients by Reader 1 and 5% by Reader 2. In MS, Dawson's fingers were seen in 92.5% patients by Reader 1 and 77.5% by Reader 2. The differences in periventricular patterns and Dawson's finger detection between NMOsd and MS were highly significant (*P* < 0.001). * Conclusions*. Dawson's fingers and “jagged-bordered” periventricular hyperintensities are typical of MS and almost never seen in NMOsd, which suggests a practical method for differentiating the two diseases.

## 1. Introduction

Neuromyelitis optica (NMO) was originally conceived as a disease with predominantly opticospinal predilection, but recent studies revealed brain involvement on MRI in up to 80% of patients [1]. Some brain lesions of NMO have distinct enhancement patterns [2, 3] and lesion morphology [4, 5], but no specific set of radiologic criteria has been validated for NMO.

Periventricular lesions have been reported in NMO [1, 6–8] and linear anterior periventricular linear lesions have been noted to be characteristic of NMO [8]. In MS, periventricular hyperintensities are common and radial callosal lesions—“Dawson's fingers”—are a helpful radiologic clue to this diagnosis. Dawson's fingers are elongated, flame-shaped, hyperintense lesions best seen on sagittal FLAIR images. They are oriented along subependymal veins and thus are perpendicular to the walls of lateral ventricles [9, 10]. Pathologically, Dawson's fingers correspond to areas of perivenous inflammation. We hypothesized that radiographic evidence of perivenous inflammation (Dawson's fingers or “jagged-bordered” hyperintense signal) is absent or rare in NMO and that examination of periventricular white matter may therefore help to differentiate NMOsd from MS.

## 2. Materials and Methods

In the NMOsd group, we included only patients with a history of recurrent optic neuritis and/or longitudinally extensive transverse myelitis and seropositivity for anti-AQP4 antibody. All adult NMOsd patients fulfilling the above criteria, who were referred by the NYU MS Center physicians (IK, JH) to the NYU Department of Radiology for brain MRI examination from January 2007 to February 2012, were included in the study. We excluded patients without the MR sequences necessary for the radiological analysis or history of relapse within 8 weeks of MRI. NMOsd subjects were group frequency matched with their two nearest alphabetical neighbours from the NYU MS Clinic who had diagnosis of MS (2010 McDonald criteria) and similar age (±2 years) and disease duration (±2 years) at the time of brain MRI.

The study was approved by institutional review board of the NYU School of Medicine.

### 2.1. Magnetic Resonance Imaging

All patients underwent MRI of the brain with a 3-T scanner (Trio; Siemens, Erlangen, Germany) using an eight-channel phased-array head coil. We analyzed axial T2-weighted sequence (repetition time msec/echo time msec, 6200/97; field of view, 220 mm; matrix, 320 × 260; section thickness, 5 mm); axial fluid-attenuated inversion recovery (FLAIR) sequence (9000/87; inversion time, 2500 msec; field of view, 220 mm; matrix, 320 × 240; 5-mm section thickness); and sagittal FLAIR sequence (9000/98; inversion time, 2500 msec; field of view, 260 mm; matrix, 320 × 224; 5-mm section thickness).

### 2.2. Image Analysis

**Figure 1:**
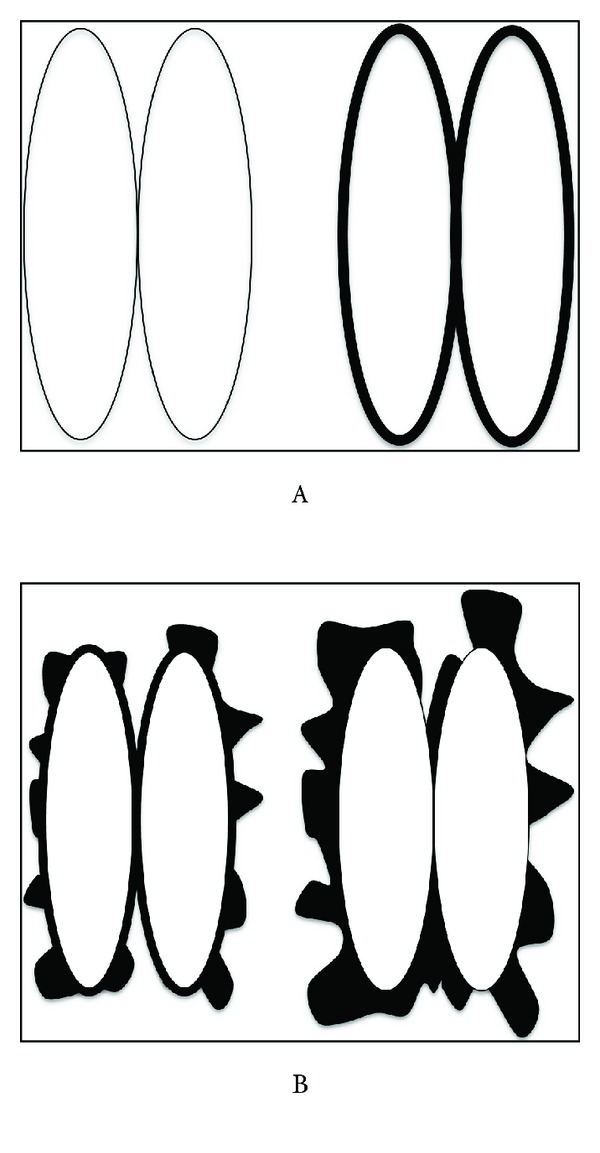
The paired ovals represent axial cross-section through lateral ventricles. Type A—no lesions on axial FLAIR (left) or smooth-bordered periventricular linear hyperintensity (right). No outpouching allowed. Type B—distinct “outpouching” periventricular lesions (left) or diffuse confluent, jagged-border hyperintensity (right).

Image analysis was performed independently by two neuroradiologists (ER and JPL) with special expertise in the field of inflammatory demyelinating diseases, who were blinded to the clinical diagnosis. The order of patients analysed was randomised. All subjects were evaluated for periventricular lesion pattern and presence of Dawson's fingers. Two periventricular lesional patterns were defined on the axial FLAIR images, as exemplified in Figure 1: Type A = no lesions or smooth periventricular linear hyperintensity, either focal or diffuse (i.e. no outpouching allowed); Type B = discrete or diffuse periventricular lesions (lesions appear to have “jagged borders”). This division was chosen in conformity with our hypothesis that perivenous inflammation is absent in NMO. Every patient was classified into either of the two pattern types. Dawson's fingers were evaluated on the sagittal FLAIR sequence. They were defined as ovoid, ≥3 mm in longest dimension, with their long axis oriented perpendicularly to the ventricular wall on the parasagittal images (caudocranial direction) [10].

### 2.3. Statistics

Group comparisons were carried out with unpaired *t*-test for continuous parametric data and Fisher's exact test or Chi square test for categorical data. We calculated the sensitivity, specificity, positive predictive value, and negative predictive values for both readers and both parameters. Cohen's kappa (*κ*) was used to evaluate for interobserver reliability between the two readers for the evaluated parameters. A *P* value < 0.05 was considered significant, and all comparisons were calculated using a two-tailed significance level. SPSS 17.0 was employed for statistical analyses.

## 3. Results

Twenty NMOsd patients and forty age- and disease duration-group frequency-matched MS patients were included in the study. Demographic and disease-related characteristics of the two groups are shown in Table 1. There were no statistical differences between the two groups with respect to age (*P* = 0.52) or disease duration (*P* = 0.42).

### 3.1. Periventricular Lesional Patterns

Periventricular hyperintensities were observed on axial FLAIR in 11/20 (55%) of NMOsd patients by both Readers 1 and 2 and 39/40 MS (98%) patients by both Readers. Type A pattern was noted in only 2/40 (5%) MS patients according to either Readers and in 17/20 (85%) of NMOsd patients according to Reader 1 and 16/20 (80%) of NMOsd patients according to Reader 2. Fisher's exact test demonstrated a statistically significant (*P* < 0.001) correlation between type A pattern and diagnosis of NMOsd, and between type B pattern and diagnosis of MS.

Sensitivity and specificity of Type A lesion pattern for NMOsd were 0.85 and 0.95, for Reader 1 and 0.80 and 0.95 for Reader 2. Positive and negative predictive values of Type A lesion pattern for NMOsd were 0.89 and 0.93 for Reader 1, and 0.93 and 0.90 for Reader 2.

**Figure 2:**
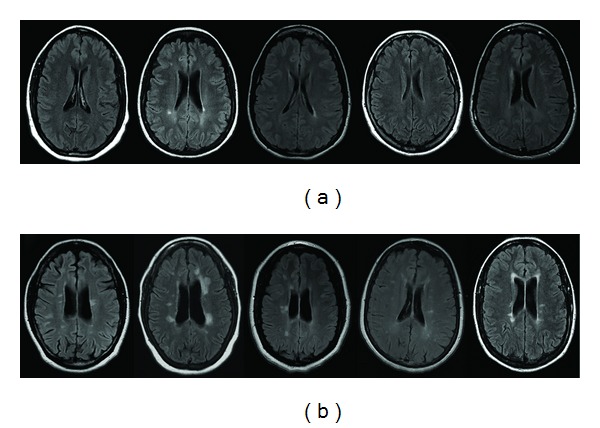
Patterns of the periventricular lesions on axial FLAIR images. Five representative patients with NMO are shown in (a): periventricular white matter is either devoid of lesions or contains smooth periventricular linear hyperintensity (with the exception of single patient). In contrast, five representative MS patients (b) demonstrate periventricular lesional patterns B, more compatible with focal perivenous inflammation.

Figure 2 shows examples of periventricular findings in NMOsd (a) and MS (b) on axial FLAIR.

### 3.2. Dawson's Fingers

Dawson's fingers were present in 37/40 (93%) MS patients according to Reader 1 and in 31/40 (78%) patients according to Reader 2. Dawson's fingers were reported in 1/20 (5%) NMOsd patient by Reader 2, and none by Reader 1. Both readers found Dawson's fingers to be significantly more frequent in MS patients compared to NMOsd patients (Fisher's Exact Test, *P* < 0.0001).

Sensitivity and specificity of absence of Dawson's fingers for NMOsd diagnosis were, respectively, 1 and 0.93 for Reader 1, and 0.95 and 0.78 for Reader 2. Positive and negative predictive values of absent Dawson's fingers for NMOsd were, respectively, 0.87 and 1 for Reader 1 and 0.68 and 0.97 for Reader 2.

**Figure 3:**
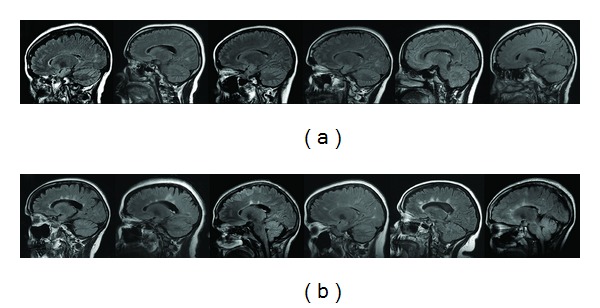
Evaluation of FLAIR sagittal images. Patients with NMO are shown in (a): Dawson's fingers are absent. In contrast, most MS patients (b) demonstrate elongated, flame-shaped lesions oriented along the course of deep medullary veins, compatible with perivenous inflammation.

Figure 3 shows examples of periventricular findings in NMOsd (a) and MS (b) on Sagittal FLAIR.

### 3.3. Interobserver Analysis

The interobserver agreement in the pattern Types evaluation was excellent, with a *k* value of 0.961 (asymptotic standard error = 0.039; *P* < 0.0001). In the dichotomic evaluation regarding the presence or absence of Dawson's fingers, the interobserver agreement was very good, with a *k* value of 0.763 (asymptotic standard error = 0.083; *P* < 0.0001).

## 4. Discussion

Recent studies document radiographic involvement of the brain in NMO in over 60% of patients [4, 11] and up to 90% of AQP4-Ab seropositive NMO patients [2, 12]. The ubiquity of brain lesions in NMOsd may complicate the task of differentiating this entity from MS. The commonly used MRI criteria for space dissemination in MS do not reliably discriminate MS from NMO: 42–50% of NMO patients [13, 14] satisfy Barkhof criteria for dissemination in space [15], and as many as 71% of NMO patients satisfy Swanton criteria for MS [16]. In our series, as well, 50% of NMOsd patients fulfilled three or more Barkhof criteria for dissemination in space, as compared to 76% of MS patients.

Certain lesions are believed to be highly characteristic for NMO, but these lesions have low sensitivity for this diagnosis. For example, “NMO-specific” periependymal lesions of the third and fourth ventricles are present in only 7% of NMO patients [4], and the typical NMO lesions in the central medulla are similarly infrequent [17]. Patchy, “cloud-like” enhancement with blurred margins [2] and “pencil-thin” ependymal enhancement [3] are also highly suggestive of NMO, but are rarely seen. Callosal lesions in MS patients appear to be larger and more confluent than in NMO [5, 18], but the sensitivity and specificity of these findings are unknown. Since MS and NMOsd require different treatment, there is an urgent need to develop reliable and practical radiographic criteria for differentiating NMOsd from MS.

We hypothesized that radiologic appearance of MS and NMO periventricular lesions would differ inasmuch as pathophysiology of the two diseases is different. In MS, periventricular radial lesions, known as “Dawson's fingers,” track along the deep medullary veins that course perpendicularly to the wall of the ventricles [19, 20]. Dawson's fingers help to differentiate MS from other white matter diseases [10, 21, 22]. Presence of central venule is a hallmark of periventricular MS lesions on ultra-high-field MRI, where it is present in ~80% of lesions [23]. In this report, we demonstrate that Dawson's fingers are rarely seen in NMO (Figure 3(a)). This dovetails with the recent observation that NMO brain lesions lack central venule on ultra-high field MRI [12, 24]. On axial FLAIR images, absence of periventricular lesions or of focal or diffuse smooth periventricular linear hyperintensities is the rule in NMO. It is possible that smooth pattern is due to wide-spread AQP-4 Ab reaction to AQP4 expressed in the ependymal layer [3]. In contrast, MS patients demonstrated “jagged” periventricular hyperintensities (Figure 2(b)), a finding compatible with focal perivenous inflammation. The differences in lesion patterns in periventricular white matter are likely the result of differences in lesional pathogenesis in the two diseases.

Our patients were evaluated, on average, 7-8 years after symptom onset. We had only 5 MS cases and 5 NMOsd cases with MRI of the brain within 3 years of symptom onset. According to both readers, all early MS cases evidenced at least one Dawson's finger, while none of NMOsd did (*P* = 0.08). Difference in the percentages of Types A and B was not significant among the early MS and NMOsd cases. These preliminary results suggest that presence or absence of Dawson's fingers early on in the disease course may help in differentiating NMOsd from MS.

It should be pointed out that other diseases could also affect periventricular white matter. The high specificity and specificity of our findings apply only to the clinical scenario where clinician is trying to differentiate NMOsd from MS. Our approach should not be used for differentiation of, for example, NMOsd and leukoaraiosis. Moreover, we compared MS patients with AQP4-seropositive NMOsd patients, a group that comprises more than 80% of NMO [25]. Our findings are not necessarily generalizable to AQP4-seronegative NMO patients.

We found that absence of Dawson's fingers on sagittal FLAIR and absent, or smooth-bordered, periventricular hyperintensities on axial FLAIR strongly favour the diagnosis of NMOsd over MS, and are more helpful than Barkhof criteria for discrimination between the two diseases. It is plausible that further improvements in MR imaging technique, such as decreasing thickness of sagittal FLAIR from 5 mm, as in our study, to 1-2 mm, would permit an even clearer and earlier separation between NMOsd and MS.

## Figures and Tables

**Table 1:** Demographic and disease-related characteristics of NMOsd and MS groups.

	NMOsd	MS
Number of patients per group	20	40
Female (%)	95%	100%
Age, mean (SD) in year	51.1 (16.6)	48.2 (14.4)
Disease duration, mean (SD) in year	7.3 (4.9)	8.4 (4.6)
NMO Ab serostatus	100%	0%

NMOsd: neuromyelitis optica spectrum disorders; MS: multiple sclerosis; SD: standard deviation; NMO Ab: neuromyelitis optica antibody.
